# Ulinastatin did not reduce mortality in elderly multiple organ failure patients: a retrospective observational study in a single center ICU

**DOI:** 10.1002/ams2.304

**Published:** 2017-08-18

**Authors:** Masatoshi Uchida, Toshikazu Abe, Kazuyuki Ono, Nanako Tamiya

**Affiliations:** ^1^ Department of Emergency and Critical Care Medicine Dokkyo Medical University Tochigi Japan; ^2^ Department of Health Services Research Faculty of Medicine University of Tsukuba Tsukuba Ibaraki Japan; ^3^ Department of Emergency and Disaster Medicine Juntendo University Urayasu Hospital Urayasu Chiba Japan

**Keywords:** Multiple organ dysfunction syndrome, multiple organ failure, sepsis, systemic inflammation response syndrome, ulinastatin

## Abstract

**Aim:**

Our aim was to evaluate the effect of ulinastatin on 28‐day mortality in patients who developed multiple organ failure (MOF) related to their acute illness and were admitted to the intensive care unit (ICU).

**Methods:**

We carried out a retrospective observational study of MOF patients in a general ICU of a tertiary care hospital in Japan from January 2009 to December 2012. The primary outcome was 28‐day all‐cause mortality. Secondary outcomes were ventilator‐free days, ICU‐free days, and vasopressor‐free days at day 28. We investigated the association between ulinastatin treatment and outcomes using multivariable regression analysis.

**Results:**

A total of 212 MOF patients were included, 79 (37%) of whom received ulinastatin. The median age was 70 years (interquartile range, 60–77) and median APACHE II score was 25 (interquartile range, 19–29). Overall 28‐day mortality was 20%. There were no significant differences between the ulinastatin group and the control group in age, gender, or APACHE II score. The ulinastatin group had higher prevalence of sepsis (44% versus 22%, *P *=* *0.001). Multivariable logistic regression analysis showed that ulinastatin was not associated with 28‐day mortality (odds ratio = 1.22; 95% confidence interval, 0.54–2.79). Moreover, ulinastatin did not reduce the mortality in patients with sepsis (odds ratio = 1.92; 95% confidence interval, 0.52–7.13). However, ICU‐free days and ventilator‐free days was significantly fewer in the ulinastatin group than control group.

**Conclusions:**

In this retrospective observational study, ulinastatin was not associated with mortality in elderly patients with established MOF, although it might be related to patient's utility.

## Background

Multiple organ failure (MOF) is a syndrome in which two or more organs cannot be maintained without intervention.^1^ Previous studies suggested that unbridled systemic inflammatory response resulting from systemic cytokine release leads to systemic inflammatory response syndrome (SIRS) and MOF.^1^ It usually results from infection, injury, shock, and hypermetabolism.^2^ Even when the causes are different, SIRS manifests a similar clinical picture and is regulated by common pro‐inflammatory cytokines, such as tumor necrosis factor‐α, interleukin (IL)‐1, and IL‐6.^2^ Excessive secretion and systemic distribution of these cytokines leads to systemic activation of neutrophils and monocytes.^2^ Activated neutrophils release lysosomal enzymes including serine proteases and reactive oxidant intermediates, and these cellular products induce tissue damage and microcirculation injuries.^2^ Unbridled systemic inflammation leads to tissue injury and injured tissue becomes the new stimulus to recycle the inflammation process, even if appropriate therapy for the initial insult has been given.^2^ To break this vicious circle of inflammation, many inflammation‐directed therapies have been studied. However, most of them have failed to show survival benefit.^2^ As a result, the mortality of MOF remains high.^2^

Ulinastatin is one a serine protease inhibitor in the multifunctional Kunitz family found in human blood and urine.^3^ Basic research showed that ulinastatin had anti‐inflammatory activity through suppressing neutrophil accumulation and activation and inhibiting secretion of inflammatory cytokines.^3^ Clinical studies also reported that anti‐inflammatory activity of ulinastatin decreased the mortality of SIRS conditions, such as sepsis^4^, ^5^ and acute respiratory distress syndrome.^6^ Thus, ulinastatin would be considered a therapeutic approach for MOF. However, these clinical studies were mainly carried out in specific countries such as China and India. It is pointed out that the health care system in India is different from developed countries.^7^ In addition, little is known about ulinastatin's effects on most critically ill patients, such as those with MOF. Therefore, it is difficult to adopt these results for critically ill patients in ICUs in developed countries. Our aim was to evaluate the effect of ulinastatin on clinical outcomes among patients with MOF.

## Methods

### Ethics

The study protocol was reviewed and approved by the ethics committee of Dokkyo Medical University (Mibu, Japan). Patient consent was not required as data were collected retrospectively.

### Study design and participants

We undertook a retrospective observational study in a 10‐bed general intensive care unit (ICU) of a tertiary care hospital in Japan from January 2009 to December 2012. Patients who were diagnosed with MOF within 24 h from ICU admission were eligible for the study. The definition of MOF referred to criteria in a previous study, which included patients with MOF caused by various diseases.^8^ Further details of definitions are as follows. Multiple organ failure was defined as the presence of two or more organ failures related to their acute illness. Organ failures were defined as: (i) respiratory failure, mechanically ventilated and a PaO_2_/FiO_2_ ratio of <300; (ii) hypoperfusion, to maintain circulation, vasopressor agents (norepinephrine, epinephrine, vasopressin, or >5 μg/kg/min dopamine) required for 2 h or longer; (iii) renal dysfunction, in patients without known chronic kidney disease, serum creatinine >1.93 mg/dL, or a urine output of <500 mL/last 24 h (or 80 mL/last 4 h if a 24‐h period of observation not available), in patients with acute on chronic renal failure (predialysis), an absolute increase of >0.90 mg/dL from baseline creatinine, or a urine output of <500 mL/last 24 h (or 80 mL/last 4 h); and (iv) thrombocytopenia, platelet count of ≤50 × 10^9^/L.^8^ We excluded patients who met any of these criteria: (i) younger than 18 years; (ii) admitted after elective cardiac surgery; (iii) died within 24 h of ICU admission; (iv) a history of organ transplantation; (v) readmission to ICU within 28 days; (vi) treated with ulinastatin before ICU admission; and (vii) treated within ulinastatin <3 days.

### Data abstraction

Medical records were reviewed and data obtained by study investigators. Baseline characteristics (age, gender, and admission category), disease category, patient comorbidities, baseline Sequential Organ Failure Assessment (SOFA) score derived from values on ICU admission and Acute Physiology and Chronic Health Evaluation Survey (APACHE) II score derived from the worst values from the first 24 h in the ICU, number of failed organs, and therapeutic interventions (ulinastatin, antibiotics, corticosteroid, vasopressor, mechanical ventilation, renal replacement therapy [RRT], intra‐aortic balloon pumping, venoarterial extracorporeal membranous oxygenation [VA‐ECMO], and operation) were recorded. The exposure was i.v. administration of ulinastatin (Miracrid; Mochida Pharmaceutical, Tokyo, Japan) during their ICU stay. Ulinastatin exposure was defined as documented administration of ulinastatin at 300,000 IU/day for 3 or more days. This dosage was approved for patients with shock in Japan. The treatment duration was 3 or more days. It based on previous randomized controlled trials (RCTs).^4^, ^5^, ^6^, ^9^, ^10^, ^11^, ^12^, ^13^ All clinical decisions, including ulinastatin administration, were at the treating physician's discretion.

### Outcome measurements

The primary outcome was 28‐day all‐cause mortality. The secondary outcomes included ventilator‐free days (VFDs), ICU‐free days (ICUFDs), and vasopressor‐free days (VASFDs) at day 28. Patients who died prior to day 28 were allocated 0 for VFDs, ICUFDs, and VASFDs.

### Statistical analysis

Dichotomous variables were reported as counts and percentage, and comparisons between the two groups were carried out with Pearson's χ^2^‐test or Fisher's exact test, as appropriate. Continuous data were reported as mean and standard deviation or median and interquartile range (IQR), and compared using Student's *t*‐test or the Mann–Whitney *U*‐test, as appropriate. All statistical tests were two‐tailed and *P*‐values <0.05 were considered statistically significant. To assess the association between ulinastatin and outcomes, we carried out multivariable logistic regressions. We used ordinal logistic regression models for VFDs, ICUFDs, and VASFDs. We carefully selected covariates if the variable had statistically significant differences between the ulinastatin and control groups in univariate analysis or if it was clinically important. Sepsis among disease category, corticosteroid use, vasopressor use, RRT, and VA‐ECMO among interventions were selected for adjusted covariates because there were statistical differences between two groups. The APACHE II score was selected for adjusted covariates because of its clinical importance. We then undertook three analytical models: crude, adjusting the APACHE II score, and adjusting factors of their severity and treatment. In the final model, adjusting factors were sepsis, corticosteroid use, vasopressor use, RRT, VA‐ECMO, and APACHE II score. Then, we stratified patients with sepsis as a secondary analysis. We did not select respiratory disease among disease category for adjusted covariate, because none were given ulinastatin in respiratory disease patients. All analyses were carried out with R version 3.1.1 (R Foundation for Statistical Computing, Vienna, Austria).

## Results

**Figure 1:**
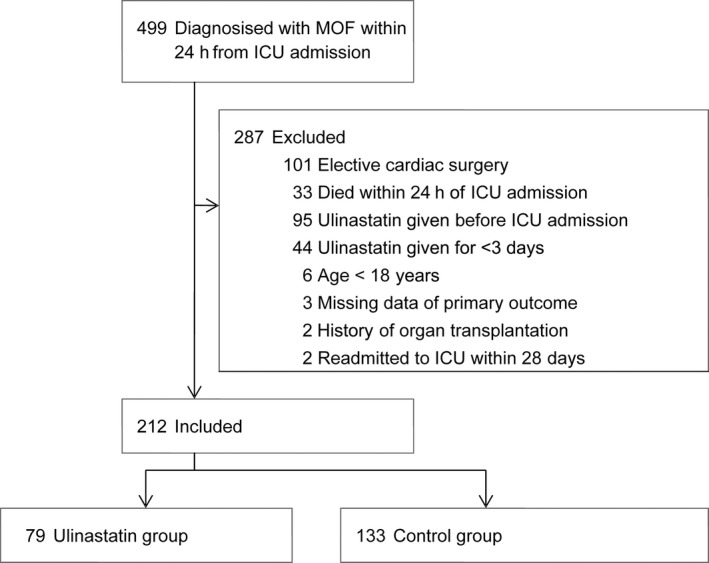
Study flow diagram of the effect of ulinastatin on mortality in elderly patients with multiple organ failure.

During the 48‐month study period, 499 patients were diagnosed with MOF within 24 h of ICU admission. Of these, 287 met exclusion criteria. Therefore, 212 patients were included in this analysis. Overall, 79/212 (37.2%) patients received ulinastatin (Fig. 1). At baseline, the median age was 70 years (IQR, 60–77), 146/212 (69%) were men, and the median APACHE II score was 25 (IQR, 19–29). Of 212 patients, 135 (64%) were aged 65 years and older, 206 (97%) received mechanical ventilation, and 205 (97%) were on vasopressors. Baseline characteristics and therapeutic interventions between the ulinastatin group and the control group are shown in Table 1. There were no significant differences between the two groups in age, gender, APACHE II score, or SOFA score. The ulinastatin group had a higher prevalence of sepsis (35/79 [44%] versus 29/133 [22%], *P* = 0.001), and they were more likely to receive corticosteroids (37/79 [47%] versus 41/133 [31%], *P* = 0.027), vasopressors (79/79 [100%] versus 126/133 [95%], *P* = 0.048), RRT (43/79 [54%] versus 49/133 [37%], *P* = 0.015), and VA‐ECMO (18/79 [23%] versus 8/133 [6.0%], *P* = 0.001). The overall 28‐day mortality was 20.3% (43/212). Univariate analyses of patient outcomes are shown in Table 2. In terms of 28‐day mortality, there was no significant difference between the ulinastatin group and the control group (20/79 [25%] versus 23/133 [20%], *P* = 0.163). The VFDs, ICUFDs, and VASFDs were significantly fewer in the ulinastatin group. In logistic regression after adjusting for APACHE II scores, there was also no significant difference in mortality (odds ratio [OR] = 1.59; 95% confidence interval [CI], 0.79–3.21). The result was similar in the final model (OR = 1.22; 95% CI, 0.54–2.79) (Table 3). Moreover, ulinastatin did not reduce the mortality in patients with sepsis (OR = 1.92; 95% CI, 0.52–7.13). After multivariable adjustment, ICUFDs and VFDs remained significantly fewer in the ulinastatin group (Table 3). Although VASFDs did not show a significant difference in the final model, there was a trend toward fewer in the ulinastatin group.

**Table 1:** Baseline characteristics of patients with multiple organ failure and ulinastatin treatment

Characteristics	Ulinastatin group (*n* = 79)	Control group (*n* = 133)	*P*‐value
Age, years, median (range)^a^	69.00 (20.00, 86.00)	70.00 (18.00, 88.00)	0.255
Age >65 years, *n* (%)	48 (60.8)	87 (65.4)	0.555
Male sex, *n* (%)	51 (64.6)	95 (71.4)	0.296
APACHE II score, median (IQR)^a^	25.00 (20.00, 30.00)	24.00 (19.00, 29.00)	0.370
SOFA score, median (IQR)^a^	9.00 (7.00, 11.00)	9.00 (7.00, 11.00)	0.886
Admission category, *n* (%)			
Medical	56 (70.9)	92 (69.2)	0.792
Elective surgery	1 (1.3)	2 (1.5)	1.000
Emergency surgery	22 (27.8)	39 (29.3)	0.819
Pre‐existing condition, *n* (%)			
Liver cirrhosis	2 (2.5)	4 (3.0)	1.000
Congestive heart failure	2 (2.5)	0 (0.0)	0.138
Chronic dialysis	7 (8.9)	13 (9.8)	0.826
Immunodeficiency	4 (5.1)	16 (12.0)	0.093
Disease category, *n* (%)			
Cardiovascular or vascular disorder	36 (45.6)	71 (53.4)	0.271
Respiratory disorder	0 (0.0)	16 (12.0)	0.001
Gastrointestinal disorder	4 (5.1)	5 (3.8)	0.730
Neurologic disorder	0 (0.0)	3 (2.3)	0.295
Sepsis	35 (44.3)	29 (21.8)	<0.001
Trauma	2 (2.5)	0 (0.0)	0.138
Metabolic disorder	0 (0.0)	1 (0.8)	1.000
Hematologic disorder	0 (0.0)	1 (0.8)	1.000
Renal disorder	0 (0.0)	3 (2.3)	0.295
Other	2 (2.5)	4 (3.0)	1.000
Post cardiac arrest, *n* (%)	14 (17.7)	16 (12.0)	0.250
Organ failure, *n* (%)			
Respiratory failure	74 (93.7)	121 (91.0)	0.485
Hypoperfusion	72 (91.1)	120 (90.2)	0.826
Renal dysfunction	35 (44.3)	48 (36.1)	0.236
Thrombocytopenia	22 (27.8)	28 (21.1)	0.260
No. of failed organs, *n* (%)			
2	43 (54.4)	89 (66.9)	0.070
3	27 (34.2)	34 (25.6)	0.180
4	9 (11.4)	10 (7.5)	0.340
Interventions, *n* (%)			
Antibiotics	79 (100.0)	129 (97.0)	0.120
Corticosteroid	37 (46.8)	41 (30.8)	0.019
Vasopressor	79 (100.0)	126 (94.7)	0.038
Mechanical ventilation	79 (100.0)	127 (95.5)	0.086
Renal replacement therapy	43 (54.4)	49 (36.8)	0.012
IABP	27 (34.2)	33 (24.8)	0.143
VA‐ECMO	18 (22.8)	8 (6.0)	<0.001
Operation	36 (45.6)	48 (36.1)	0.172

a Mann–Whitney *U*‐test.

APAHCE II, acute physiology and chronic health evaluation II; IABP, intra‐aortic balloon pumping; IQR, interquartile range; SOFA, sequential organ failure assessment; VA‐ECMO, venoarterial extracorporeal membranous oxygenation.

**Table 2:** Outcomes in patients with multiple organ failure and ulinastatin treatment

	Ulinastatin group (*n* = 79)	Control group (*n* = 133)	*P*‐value
Death at 28 days, *n* (%)^a^	20 (25.3)	23 (17.3)	0.163
ICU‐free days^b^			
Median	12	19	<0.001
Interquartile range	0–18.5	10–22	
Vasopressor‐free days^b^			
Median	16	21	0.009
Interquartile range	0–23	8–25	
Ventilator‐free days^b^			
Median	9	19	<0.001
Interquartile range	0–17	0–24	

a χ^2^‐test.

b Mann–Whitney *U*‐test.

ICU, intensive care unit.

**Table 3:** Analyses of the relationship between ulinastatin treatment and outcomes

Outcome	OR	95% CI of OR	*P*‐value
Primary outcome: 28‐day all‐cause mortality^a^			
Crude	1.62	0.82–3.19	0.162
Adjusted for APACHE II score	1.59	0.79–3.21	0.195
Multivariable adjusted^b^	1.22	0.54–2.79	0.632
Secondary outcomes			
ICU‐free days^c^			
Crude	0.31	0.19–0.52	<0.001
Multivariable adjusted^b^	0.44	0.25–0.75	0.003
Ventilator‐free days^c^			
Crude	0.42	0.25–0.69	<0.001
Multivariable adjusted^b^	0.49	0.28–0.84	0.010
Vasopressor‐free days^c^			
Crude	0.50	0.31–0.82	0.006
Multivariable adjusted^b^	0.66	0.38–1.15	0.143

a Logistic regression.

b Adjusted for APACHE II score, sepsis, corticosteroid use, vasopressor use, renal replacement therapy, and venoarterial extracorporeal membranous oxygenation.

c Ordinal logistic regression.

APACHE II, acute physiology and chronic health evaluation II; CI, confidence interval; ICU, intensive care unit; OR, odds ratio.

## Discussion

### Key findings

In this retrospective observational cohort study, we evaluated the association between ulinastatin and clinical outcomes in severely ill established MOF patients. We found that ulinastatin was not associated with 28‐day mortality. This finding did not change in the subgroup analysis of sepsis patients. The VFDs, ICUFDs, and VASFDs in the ulinastatin group were fewer than in the control group. In this study, treatment with ulinastatin was not based on a specific criteria. Although ulinastatin is approved for shock due to trauma, infection, burn, hemorrhage, acute hypoperfusion, and pancreatitis in Japan, there is no robust indication for critically ill patients. Consequently, treatment with ulinastatin was based on the treating physician's discretion in the studied institution. As a result, there were some differences between the ulinastatin and control groups. There were no statistically significant differences in severity score, but patients in the ulinastatin group were more likely to receive invasive organ support therapies, such as RRT and VA‐ECMO, than patients in the control group. Similarly, corticosteroid and vasopressor were more used more often in the ulinastatin group. Generally, corticosteroid was used for refractory septic shock patients. Furthermore, the ulinastatin group had a higher prevalence of sepsis. These findings suggested that physicians tend to prescribe ulinastatin for more severe patients, such as those with more severe organ failure or refractory septic shock. However, the results were not changed after adjustment for these imbalances among severity, therapeutic interventions, and disease category. That being said, the effects of ulinastatin might have been underestimated, because there was no significant difference in mortality between the two groups, even though patients in the ulinastatin group had more severe health conditions.

### Relationship to previous studies

The anti‐inflammatory activities, organ protective effect, and clinical effectiveness of ulinastatin in several SIRS conditions have been investigated in many animal studies^14^, ^15^, ^16^ and clinical studies.^5^, ^10^, ^13^, ^17^, ^18^, ^19^ Based on these results, we hypothesized that ulinastatin would have beneficial effects on MOF patients. However, ulinastatin treatment was not associated with a favorable outcome in our study. There were some reasons. First, even though ulinastatin affects the plasma concentration of cytokines and inflammatory mediators, it may not affect mortality. Wu *et al*.^11^ undertook an RCT of the effects of ulinastatin for patients with severe sepsis in China. There was no significant difference in 28‐day mortality (control group, 18.2% versus ulinastatin group, 20.2%, *P *>* *0.05) although ulinastatin significantly reduced inflammatory mediators in their study. Studies of patients undergoing cardiac surgery with cardiopulmonary bypass reported similar results in two meta‐analyses.^20^, ^21^ This discrepancy between effects for inflammatory mediators and clinical effectiveness was also observed in other anti‐inflammatory therapy such as drotrecogin, activated protein C.^22^, ^23^ Effects on inflammatory mediators may not be directly linked to clinical benefits in any situation. Second, ulinastatin might reduce mortality in relatively young and mildly ill patients. An RCT in severe sepsis patients^5^ showed that the ulinastatin group had lower 28‐day mortality (4/55 [7.3%] of ulinastatin group versus 12/59 [20.3%] of control group, *P *=* *0.045) and more VFDs (19.4 ± 10.6 versus 10.2 ± 12.5, *P *=* *0.019). In that study, patients aged more than 60 years and with a platelet count <30 × 10^9^/L were excluded. Consequently, included patients’ mean age was 37.1 years and mean APACHE II score was 13.4. Moreover, 74/119 (62.2%) patients had single organ failure. Another RCT in SIRS patients in China^10^ observed that ulinastatin treatment reduced mortality (1/30 [3.3%] in ulinastatin group versus 6/30 [20%] in control group, *P *<* *0.05) and the occurrence rate of MODS (multiple organ dysfunction syndrome) (3/30 (10%) versus 11/30 (36.7%), respectively, *P *<* *0.05). In that study, patients’ mean age was 43 years. In another RCT for pancreatitis patients in India,^13^ ulinastatin treatment reduced mortality (1/35 (2.8%) in ulinastatin group versus 6/32 (18.7%) in placebo group, *P *=* *0.048) in severe pancreatitis patients. In that study, patients aged more than 70 years were excluded. As a result, the mean age of severe pancreatitis was 42.2 years and the median APACHE II score was 11 in the ulinastatin group (versus 13 in the control group). In short, studies that have reported ulinastatin's favorable effects included relatively young and mild ill patients compared to ours (median age, 70 years; median APACHE II score, 25). Previous studies suggested that ulinastatin's favorable effects for SIRS patients was caused by its anti‐inflammatory features (to decrease pro‐inflammatory mediators such as tumor necrosis factor‐α and to increase anti‐inflammatory mediators such as IL‐10).^10^, ^24^, ^25^ Meanwhile, it is said that the individual immune response is different between young and elderly patients.^26^ Among elderly patients and those with numerous comorbidities, absent hyper‐inflammatory response is common and patients rapidly develop the anti‐inflammatory state.^26^ For these patients, they might be already in an immunosuppressive state when they develop MOF. This could explain why ulinastatin was not effective for our study cohort. Finally, therapy with ulinastatin alone might not be effective. Several studies^9^, ^24^, ^25^, ^27^ reported survival benefit from ulinastatin combined with thymosin α1. Combination therapy might be effective.

### Significance and implications

To the best of our knowledge, this is the first study to investigate the effects of ulinastatin treatment on established MOF. As stated above, our cohort included the most severely ill and elderly patients, compared with previous studies. Our studied patients were very specific. However, the number of elderly patients in the ICU are increasing and their poor outcomes have been reported.^28^ We think it is important to report that ulinastatin would not have significant effects for elderly and critically ill patients. Again, ulinastatin might have beneficial effects for relatively young and mildly ill patients, based on previous studies.^5^, ^10^, ^13^ Several animal and human studies reported that ulinastatin could have an organ‐protective effect.^15^, ^29^, ^30^ Hence, ulinastatin may work as “prevention” rather than “treatment” for organ dysfunction. It would be important to detect the patients who might develop organ dysfunction in the early phase of disease and to give appropriate intervention.

### Limitations

Our study has potential limitations. First, MOF is a well‐known clinical entity but there is no widely used definition. For example, liver dysfunction and dysfunction of the central nervous system were not included in the criteria of MOF we used. Therefore, our results could not be adopted for patients with MOF based on different criteria. Second, this study was retrospective. Therefore, there were several differences between the ulinastatin and control groups. As we stated before, ulinastatin was administered for more severity ill patients. This indication difference whether administrate ulinastatin or not might have led to selection bias. In particular, significant differences in secondary outcomes might have been the result of this bias. We were aware of this bias based on patients’ severity and disease category, but we adjusted it using multivariable analysis. However, there might have been uncontrolled confounders. Moreover, because of the nature of the retrospective observational study design we cannot establish causality.

## Conclusions

This retrospective study suggested that ulinastatin treatment should not be associated with favorable outcomes in elderly patients with established MOF.

## Disclosure

Approval of the research protocol: This study protocol was reviewed and approved by the ethics committee of Dokkyo Medical University, which does not require informed consent from patients for observational studies using anonymous data such as those used in this study.

Informed consent: The ethics committee of Dokkyo Medical University did not required informed consent from patients for observational studies using anonymous data such as those used in this study.

Registry and the Registration No. of the study/Trial: Not applicable.

Conflict of interest: The authors have no conflict of interest.
